# Transient Hepatic Overexpression of Insulin-Like Growth Factor 2 Induces Free Cholesterol and Lipid Droplet Formation

**DOI:** 10.3389/fphys.2016.00147

**Published:** 2016-04-25

**Authors:** Sonja M. Kessler, Stephan Laggai, Elien Van Wonterghem, Katja Gemperlein, Rolf Müller, Johannes Haybaeck, Roosmarijn E. Vandenbroucke, Manfred Ogris, Claude Libert, Alexandra K. Kiemer

**Affiliations:** ^1^Department of Pharmacy, Pharmaceutical Biology, Saarland UniversitySaarbrücken, Germany; ^2^Inflammation Research Center, VIBGhent, Belgium; ^3^Department of Biomedical Molecular Biology, Ghent UniversityGhent, Belgium; ^4^Department of Microbial Natural Products, Helmholtz Institute for Pharmaceutical Research Saarland, Helmholtz Centre for Infection Research and Pharmaceutical Biotechnology, Saarland UniversitySaarbrücken, Germany; ^5^Institute of Pathology, Medical University of GrazGraz, Austria; ^6^Department of Pharmaceutical Chemistry, University of ViennaVienna, Austria

**Keywords:** insulin-like growth factor 2 (IGF2), NASH, hydrodynamic gene delivery, fatty liver, lipid droplets

## Abstract

Although insulin-like growth factor 2 (IGF2) has been reported to be overexpressed in steatosis and steatohepatitis, a causal role of IGF2 in steatosis development remains elusive. Aim of our study was to decipher the role of IGF2 in steatosis development. Hydrodynamic gene delivery of an *Igf2* plasmid used for transient Igf2 overexpression employing codon-optimized plasmid DNA resulted in a strong induction of hepatic *Igf2* expression. The exogenously delivered Igf2 had no influence on endogenous *Igf2* expression. The downstream kinase AKT was activated in Igf2 animals. Decreased ALT levels mirrored the cytoprotective effect of IGF2. Serum cholesterol was increased and sulfo-phospho-vanillin colorimetric assay confirmed lipid accumulation in Igf2-livers while no signs of inflammation were observed. Interestingly, hepatic cholesterol and phospholipids, determined by thin layer chromatography, and free cholesterol by filipin staining, were specifically increased. Lipid droplet (LD) size was not changed, but their number was significantly elevated. Furthermore, free cholesterol, which can be stored in LDs and has been reported to be critical for steatosis progression, was elevated in Igf2 overexpressing mice. Accordingly, *Hmgcr/HmgCoAR* was upregulated. To have a closer look at *de novo* lipid synthesis we investigated expression of the lipogenic transcription factor SREBF1 and its target genes. SREBF1 was induced and also SREBF1 target genes were slightly upregulated. Interestingly, the expression of *Cpt1a*, which is responsible for mitochondrial fatty acid oxidation, was induced. Hepatic IGF2 expression induces a fatty liver, characterized by increased cholesterol and phospholipids leading to accumulation of LDs. We therefore suggest a causal role for IGF2 in hepatic lipid accumulation.

## Introduction

Lipid accumulation is a major feature accompanying all etiologies of chronic hepatitis. Hepatitis is provoked by viral factors, alcoholic, or non-alcoholic steatohepatitis. Chronic hepatitis represents a risk factor for the development of hepatocellular carcinoma (HCC), which is the second leading cause of cancer related death in men (Jemal et al., 2011).

The hepatitis C virus (HCV) core protein was shown to upregulate insulin-like growth factor 2 (IGF2) expression (Liu et al., 2005; Nguyen et al., 2006). *IGF2* has been reported to be overexpressed in advanced stages of liver disease, i.e., cirrhosis and HCC (Iizuka et al., 2002; Sedlaczek et al., 2003; Couvert et al., 2008; Kessler et al., 2013). *Igf2* transgenic mice carrying a mostly urinary promoter show a higher frequency of diverse malignancies (Rogler et al., 1994). Interestingly, genetic variants of the *IGF2* gene were correlated to obesity, obesity-associated hypertension, and intramuscular fat (Faienza et al., 2010; Aslan et al., 2012; Deodati et al., 2013). Although *IGF2* has been reported to be overexpressed in steatosis and steatohepatitis (Chiappini et al., 2006; Tybl et al., 2011; De Minicis et al., 2014), a causal role of IGF2 in steatosis development remains elusive. IGF2 signaling can be involved in the transition of steatosis and steatohepatitis to HCC due to downregulation of the tumor suppressor PTEN by IGF2 (Horie et al., 2004; Vinciguerra et al., 2008). On the other hand, IGF1 levels have been described to be downregulated in steatotic patients (Völzke et al., 2009; Mallea-Gil et al., 2012; Cianfarani et al., 2014).

*Igf2* transgenic mice show fetal overgrowth and neonatal lethality (Sun et al., 1997) and can therefore not be used to study the role of IGF2 in steatosis. Hydrodynamic delivery displays a simple and effective method for *in vivo* gene delivery (Liu et al., 1999; Suda et al., 2006). It was demonstrated in mice that DNA, RNA, and proteins but also small molecules can be delivered to hepatocytes through tail vein injection (Herweijer and Wolff, 2006; Suda and Liu, 2007). Over 99% of gene expression following a hydrodynamic tail vein delivery of plasmid DNA occurs in the liver (Zhou et al., 2010). This might be due to the unique structure of the liver sinusoids (Suda et al., 2006).

In this study we present that transient IGF2 overexpression leads to hepatic lipid accumulation.

## Materials and methods

### Animals and hydrodynamic gene delivery

Twenty-one six-week-old male C57BL/6JRj mice were purchased from Janvier Labs (Saint-Berthevin Cedex, France) and were directly delivered to their final destination. The mice were adapted to the new environment for 1 week and housed with 4–6 mice/cage in a specific pathogen-free animal facility with free access to food and water and with a 14-h light/10-h dark cycle before proceeding with the hydrodynamic injection. Seven-week-old C57BL/6JRj mice were rapidly injected (5 s) *via* the tail vein with a volume corresponding to 10% of their body weight containing plasmid DNA at a concentration of 12.5 μg/ml in sterile, endotoxin free PBS. This injection method leads to transient hepatic expression of the plasmids (Pinheiro et al., 2013). After 7 days the animals were sacrificed, liver weights were estimated, and samples were collected. The experiment was conducted in accordance with the regulations for animal experiments at the University of Ghent.

### Plasmids

**Figure 1:**
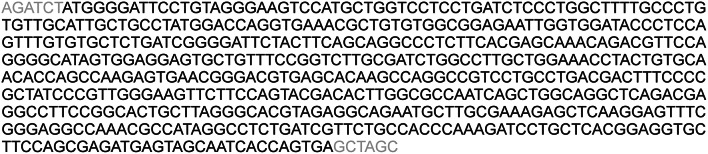
**Plasmid sequence and vector maps**. The sequence of the *Igf2* insert is shown. Restriction sites are highlighted in gray.

The backbone of a pCpG-hCMV-EF1α-LucSH (luc luciferase, sh neomycin resistance) plasmid, which has been shown to induce high and stable expression levels of the luciferase insert (Magnusson et al., 2011), was used for the generation of the pCpG-hCMV-EF1α-Igf2 plasmid (Igf2). The protein coding murine *Igf2* cDNA sequence was chosen according to published sequence data (NM_010514.3, NM_001122736.1, NM_001122737.1), optimized for codon usage, flanked by *Bgl*II and *Nhe*I restriction sites, synthesized by Eurofins Genomics (Ebersberg, Germany), and finally cloned into the pCpG-hCMV-EF1α backbone. The optimized insert sequence corresponds to the published murine IGF2 protein amino acid sequence data (NP_001116209.1, NP_001116208.1). All procedures were performed according to published methods (Magnusson et al., 2011; Su et al., 2013). Plasmid purification was done with the EndoFree Plasmid Giga Kit from Qiagen (12391, Hilden, Germany) under sterile, endotoxin free conditions. The final plasmid sequence was validated by sequencing (Eurofins Genomics). Ten mice were injected with the Luc plasmid as control animals (co) and 11 mice were injected with the *Igf2* plasmid (Igf2). The insert sequence is provided in Figure 1. Plasmids were deposited at Addgene (http://www.addgene.org; plasmid 67933, plasmid 67934).

### Serum parameters

Two days after injection blood glucose was determined by a BGStar glucometer (MDSS GmbH, Hannover, Germany). After 7 days animals were sacrificed and serum was collected. Serum levels of high density lipoprotein (HDL), triglycerides, cholesterol, aspartate aminotransferase (AST), and alanine aminotransferase (ALT) were measured at the “Zentrallabor des Universitätsklinikums des Saarlandes” (University of Saarland, Homburg, Germany).

### Real-time RT-PCR

Real-time RT-PCR was performed as recently reported (Laggai et al., 2014). Real-time RT-PCR was performed in a CFX96 cycler (Bio-Rad, Munich, Germany) with 5 × HOT FIREPol® EvaGreen® qPCR Mix Plus (Solis BioDyne, Tartu, Estonia). All samples were measured in triplicate. Efficiency was determined for each experiment using a cDNA dilution series with a standard dilution series as described previously (Kiemer et al., 2009). The relative gene expression was normalized to *18S* mRNA values. Details on primer sequences and conditions can be found in Table 1.

**Table 1:** **Primer sequences and conditions**.

**Gene**	**Forward primer sequence 5′–3′**	**Reverse primer sequence 5′–3′**	**Amplicon size [bp]**	**Gene bank accession number**	**Primer [nM]**	**Annealing Temp. [°C]**
18s	AGGTCTGTGATGCCCTTAGA	GAATGGGGTTCAACGGGTTA	109	NR_003278.3	250	61
Acaca	TGGAGCTAAACCAGCACTCC	GTGTATCTGAGCTGACGGAGG	141	NM_133360.2	200	60
Cpt1a	CTCAGTGGGAGCGACTCTTCA	GGCCTCTGTGGTACACGACAA	105	NM_013495.2	250	60
Elovl6	ACAATGGACCTGTCAGCAAA	GTACCAGTGCAGGAAGATCAGT	119	NM_130450.2	100	60
Emr1	CTTTGGCTATGGGCTTCCAGTC	GCAAGGAGGACAGAGTTTATCGTG	165	NM_010130	150	60
exogenous Igf2	CACGAGCAAACAGACGTTCC	CTCACGTCCCGTTCACTCTT	100	−	200	60
Fasn	ATCCTGGAACGAGAACACGATCT	AGAGACGTGTCACTCCTGGACTT	140	NM_007988.3	150	60
Gck	GGACTCCACACCCCACAAAT	GCTGTCTCACTGGCTGACTT	113	NM_010292.4	250	60
Hmgcr	ATCCAGGAGCGAACCAAGAGAG	CAGAAGCCCCAAGCACAAAC	99	NM_008255.2	250	60
IGF2	GGAAGTCGATGTTGGTGCTTCTC	CGAACAGACAAACTGAAGCGTGT	121	NM_010514.3	250	60
Mlxipl	CTGGGGACCTAAACAGGAGC	GAAGCCACCCTATAGCTCCC	166	NM_021455.4	250	60
Nr1h3	CCGACAGAGCTTCGTCC	CCCACAGACACTGCACAG	81	NM_013839.4	200	60
Pklr	GCTCTGGCCCTGGATCTTTA	CTGGCACGTCTCAGGTATCC	96	NM_013631.2	200	60
Ppara	CCTTCCCTGTGAACTGACG	CCACAGAGCGCTAAGCTGT	77	NM_001113418.1	250	60
Scd	AGATCTCCAGTTCTTACACGACCAC	CTTTCATTTCAGGACGGATGTCT	140	NM_009127.4	200	60
Srebf1c	GGCTCTGGAACAGACACTGG	GGCCCGGGAAGTCACTGT	110	NM_011480.3	100	60

### Protein isolation and analysis by western blot

Western blot of total tissue lysates was carried out as described previously (Kessler et al., 2013; Laggai et al., 2014). Anti-α-tubulin (T9026, Sigma Aldrich, Taufkirchen, Germany, 1:1000 in PBST + 5% dry milk) and anti-SREBF1 [ab3259, Abcam, Cambridge, UK, 1:200 in Rockland blocking buffer (RBB; MB-070, Gilbertsville, USA)] antibodies were incubated for 3 h at room temperature. Anti-pAKT antibody (#4060P, Cell Signaling Technology, Danvers, USA, 1:1000 in RBB) was incubated overnight at 4°C. Secondary antibodies against mouse IgG [for tubulin and SREBF1, IRDye® 800cw Conjugated Affinity Purified Anti Mouse IgG (H&L) (Goat), 926–32210, LI-COR Biosciences, Licoln, USA] and against rabbit IgG [for pAKT, 926–68071 IRDye® 680RD Goat anti-Rabbit IgG (H&L), 926–68071, LI-COR Biosciences] were diluted 1:10,000 in RBB and blots were incubated for 1.5 h at room temperature. Detection was done with a LI-COR Odyssey device (LI-COR Biosciences).

### Scharlach red staining for lipids

Cryo liver sections (5 μm; co, *n* = 4; Igf2, *n* = 4) were fixed with 4% normal buffered formalin for 2 min, treated with 50% ethanol for 3 min, stained for 3 min with 0.3% [m/v] Scharlach Red (0327.1, Roth, Karlsruhe, Germany) in 1:1 aceton/70% ethanol [v/v], rinsed with 70% ethanol and counterstained with hematoxylin (T865.2, Roth) (Simon et al., 2014a,b). Slides were embedded in the water based FluoroSafe™ mounting medium (#345789, Merck, Darmstadt, Germany). Pictures were captured by an Axio Star plus microscope coupled to an Axio Cam ICc 1 camera (both Zeiss, Feldbach, Swiss).

### Quantification of total lipids

Total lipids were quantified by sulfo-phospho-vanillin colorimetric method according to published methods (Cheng et al., 2011). Total lipids were extracted as previously published (Laggai et al., 2013). Dry lipids were dissolved in chloroform/methanol (2:1; v/v), loaded into glass vials, from which the solvent was evaporated at 90°C. After cooling the samples down to room temperature samples were incubated with 100 μl sulfuric acid (conc.) at 90°C for 20 min and afterwards cooled down to room temperature on ice. Fifty microliters of vanillin-phosphoric acid (0.2 mg vanillin per ml 17% ortho-phosphoric acid) were added, incubated for 10 min, and 100 μl of the colored solutions were transferred to 96 well plate before measurement of the absorbance at 550 nm in a Sunrise™ Basic plate reader (Tecan, Maennedorf, Switzerland).

### Quantification of lipid classes by thin layer chromatography (TLC)

Lipid extraction and TLC analysis was performed as recently described using a sulfuric acid/ethanol mixture for detection (Laggai et al., 2013). Fifteen milligrams of the freeze-dried tissue was dispersed with a mixture of hexane/2-propanol [3:2 (v/v)] for 10 min, and centrifuged at 4°C and 10,000 g for 10 min. The supernatant was dried under a nitrogen stream, redissolved in chloroform/methanol [1:1 (v/v)], and applied in equal amounts onto the TLC plates. After prewashing with a mixture of chloroform/methanol [2:1 (v/v)] TLC plates were activated at 110°C for 1 h. The samples and standard substances were applied onto the TLC plates, which were developed in chloroform/methanol/acetic acid/water [50:30:8:3 (v/v/v/v)] until the liquid phase front reached half of the plate. Then the development was stopped, the plate was dried, and then fully developed in heptane/diethyl ether/acetic acid [70:30:2 (v/v/v)]. The ratio area density of each band was quantified using the ImageJ software as described in Laggai et al. (2013).

### Fluorescence staining for lipids and unesterified cholesterol

For lipid staining, cryo liver sections (5 μm; co, *n* = 10; Igf2, *n* = 11) were fixed with 4% normal buffered formalin for 5 min, washed three times for 10 min with PBS, incubated with the highly selective fluorescent lipid dye LD540 (Astanina et al., 2015) (0.5 μg/ml in PBS) for 15 min at room temperature, washed three times with PBS for 10 min, incubated for 1 min with diaminophenylindol HCl (DAPI) (D9542, Sigma Aldrich) at a concentration of 1 μg/ml, and afterwards washed three times with PBS. For the staining of free cholesterol, we performed filipin (F4767-1 mg, Sigma Aldrich) staining according to published methods (Ioannou et al., 2013; Simon et al., 2014b). All steps were performed under exclusion of direct light. Slides were embedded in the water based FluoroSafe™ mounting medium and visualized with an inverse fluorescence microscope at 558/583 (excitation/emission) for LD540 and at 359/461 (excitation/emission) for DAPI and filipin (Axio Observer, Zeiss, Feldbach, Swiss). Five randomly selected pictures from each sample were collected.

### Determination of lipid droplet properties

Lipid droplet (LD) number, size, and area were estimated from LD540 and DAPI stained slides as described above. The five randomly selected pictures from each sample were analyzed using the “analyse particles” function of ImageJ 1.47i (Schneider et al., 2012) as described in published methods (Beller et al., 2008; Sjøbakk et al., 2013). The lipid droplets and nuclei were segmented using a manual threshold. Watershed processing was performed to separate LDs and nuclei. Measurements on the edges were excluded and lipid droplets were measured in a pixel size from one to infinity and nuclei were measured in a pixel size from fifty to infinity and both with parameters for circularity from zero to one. The total number of particles and the respective area were measured. The results were validated by the use of the ImageJ plugin Lipid Droplet Counter (Author: Samuel Moll; http://imagejdocu.tudor.lu/doku.php?id=plugin:analysis:droplet_counter:start) as previously published (Mahammad and Parmryd, 2008; Astanina et al., 2015). The LD number and the total area were normalized on the nuclei number from each picture and the mean of the five pictures per slide was estimated. Average lipid droplet size was estimated as the mean area of all single lipid droplets per picture and the five pictures per slide.

### Cholesterol and fatty acid measurement by gas chromatography-mass spectrometry (GC-MS)

Cholesterol and fatty acids were measured by GC-MS as reported recently (Simon et al., 2014b).

### Statistical analysis

Data analysis and statistics were performed with the Origin 8.6 software (OriginLab Corporation, Northampton, USA). Results are expressed as mean ± SEM. The statistical significance for normally distributed data was determined by independent two-sample *t*-test, for non-normally distributed data non-parametric Wilcoxon rank sum test was employed. *p* < 0.05 were considered as statistically significant.

## Results

### Validation of the *Igf2* hydrodynamic gene delivery and characterization of the *Igf2* model

**Figure 2:**
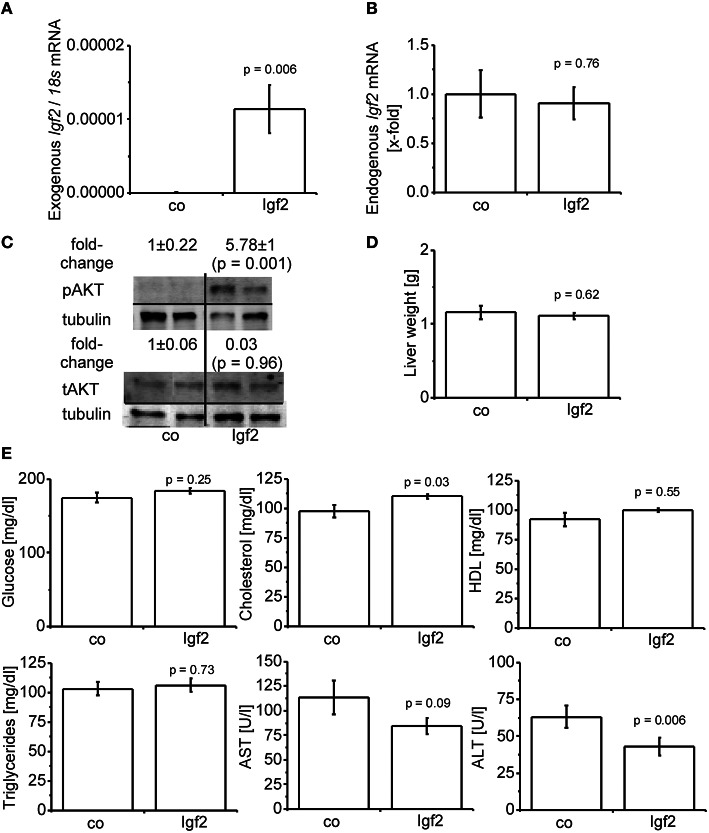
**Characterization of the IGF2 overexpression model**. **(A)** Exogenous *Igf2* mRNA after hydrodynamic gene delivery of the Igf2 plasmid (Igf2) compared to the control plasmid (co), real-time RT-PCR (co, *n* = 10; Igf2, *n* = 11, Wilcoxon rank sum test). Data were normalized to *18S* mRNA. **(B)** Endogenous *Igf2* mRNA levels, real-time RT-PCR (co, *n* = 10; Igf2, *n* = 11, independent two-sample *t*-test), were normalized to *18S* mRNA and co. **(C)** Western blot analysis of phosphorylated and total AKT protein levels (co, *n* = 8; Igf2, *n* = 9, independent two-sample *t*-test), densitometric data were normalized to α-tubulin and co. **(D)** Liver weight on day 7 after plasmid injection (co, *n* = 10; Igf2, *n* = 11, independent two-sample *t*-test). **(E)** Serum parameters 2 days (glucose) or 7 days (other parameters) after plasmid injection. Glucose and triglycerides (co, *n* = 10; Igf2, *n* = 11, independent two-sample *t*-test). Cholesterol, HDL, AST, and ALT (co, *n* = 10; Igf2, *n* = 11, Wilcoxon rank sum test). All results are presented as mean ± SEM.

Hydrodynamic gene delivery has been widely used for the overexpression of plasmid DNA and the desired product in murine livers (Zhu et al., 2006; Magnusson et al., 2011; Pinheiro et al., 2013; Timmermans et al., 2015). Concordantly, total *Igf2* mRNA was highly expressed by the injection of the *Igf2* plasmid (Figure 2A). Exogenously delivered *Igf2* had no influence on the endogenic *Igf2* mRNA levels (Figure 2B). As a marker for IGF2-induced insulin signaling the downstream anti-apoptotic effector of IGF2, AKT (Hamamura et al., 2008; Tybl et al., 2011), was phosphorylated and therefore activated (Figure 2C). The liver weight of the Igf2 injected animals was not altered after 7 days (Figure 2D), nor were the serum parameters glucose, HDL, and triglycerides (Figure 2E). Interestingly, though, serum cholesterol was increased (Kobayashi et al., 2004). Hydrodynamic gene delivery usually increases ALT and AST levels, which return to normal after 3 days. In the Igf2 injected animals ALT was decreased, and also AST showed a strong tendency of lower levels compared to animals obtaining the Luc control plasmid. Reduced liver damage is most likely linked to cytoprotective actions of IGF2 (Nielsen, 1992; O'Dell and Day, 1998). The AST/ALT ratio remained unaffected in the Igf2 injected animals compared to the controls (*p* = 0.57, co, *n* = 10; Igf2, *n* = 11, Wilcoxon rank sum test).

### *Igf2* induces steatotic histology and lipid accumulation

**Figure 3:**
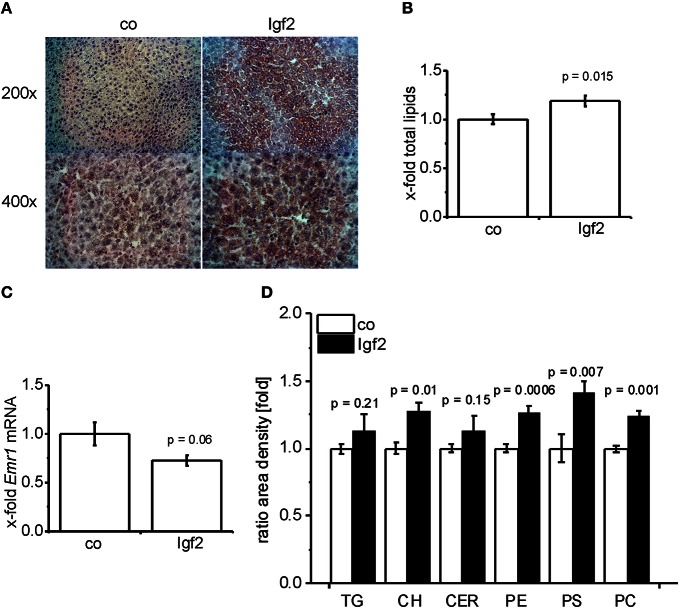
**IGF2 induces lipid accumulation without inflammation**. **(A)** Representative Scharlach Red staining for lipids (red staining) in animals injected with Igf2 plasmid (Igf2) compared to the control plasmid (co), nuclei were counterstained with hematoxylin. Original magnification was 200 × or 400 × (each group, *n* = 4). **(B)** Quantification of total lipids *via* sulfo-phospho-vanillin colorimetric assay. Igf2 animals normalized to co animals (co, *n* = 10; Igf2, *n* = 11, independent two-sample *t*-test). **(C)** mRNA levels of the macrophage marker *Emr1* (*F4/80*), real-time RT-PCR (co, *n* = 10; Igf2, *n* = 11, independent two-sample *t*-test). Data were normalized to *18S* mRNA and co. **(D)** Quantification of lipid classes by TLC: triglycerides (TG), cholesterol (CH), ceramides (CER), phosphatidylethanolamine (PE), phosphatidylserine (PS), and phosphatidylcholine (PC) in Igf2 animals normalized to co animals (co, *n* = 10; Igf2, *n* = 11, independent two-sample *t*-test). All results are presented as mean ± SEM.

Scharlach Red staining (Figure 3A) showed that IGF2 induced features of mild steatosis without any specific zonation of lipid accumulation. The colorimetric quantification of total lipids confirmed significantly increased hepatic lipid content (Figure 3B). Interestingly, signs of liver inflammation, as validated by the widely used macrophage specific marker (Khazen et al., 2005; Laggai et al., 2014). EGF-like module-containing mucin-like hormone receptor-like 1 (Emr1/F4/80) mRNA levels, were absent in the livers of Igf2 injected animals (Figure 3C). Since also human steatosis is characterized not only by quantitative, but also by qualitative changes in lipid and fatty acid composition (Puri et al., 2007, 2009), we employed TLC and GC-MS methods in order to decipher the respective composition. Interestingly, the most distinct increases were found for cholesterol and the phospholipids phosphatidylethanolamine, phosphatidylserine, and phosphatidylcholine (Figure 3D). Neither triglyceride nor ceramide levels were significantly different (Figure 3D). GC-MS analyses confirmed the increase in liver cholesterol (*p* = 0.03, co, *n* = 10; Igf2, *n* = 11, Wilcoxon rank sum test). Measurement of single fatty acids revealed a slight increase of docosahexanoic acid in Igf2 mice (7.75 ± 0.09 compared to 6.99 ± 0.18 μg/mg tissue in control animals; *p* = 0.008). All other fatty acids were not altered (data not shown).

### *Igf2* induces lipid droplet formation and free cholesterol

**Figure 4:**
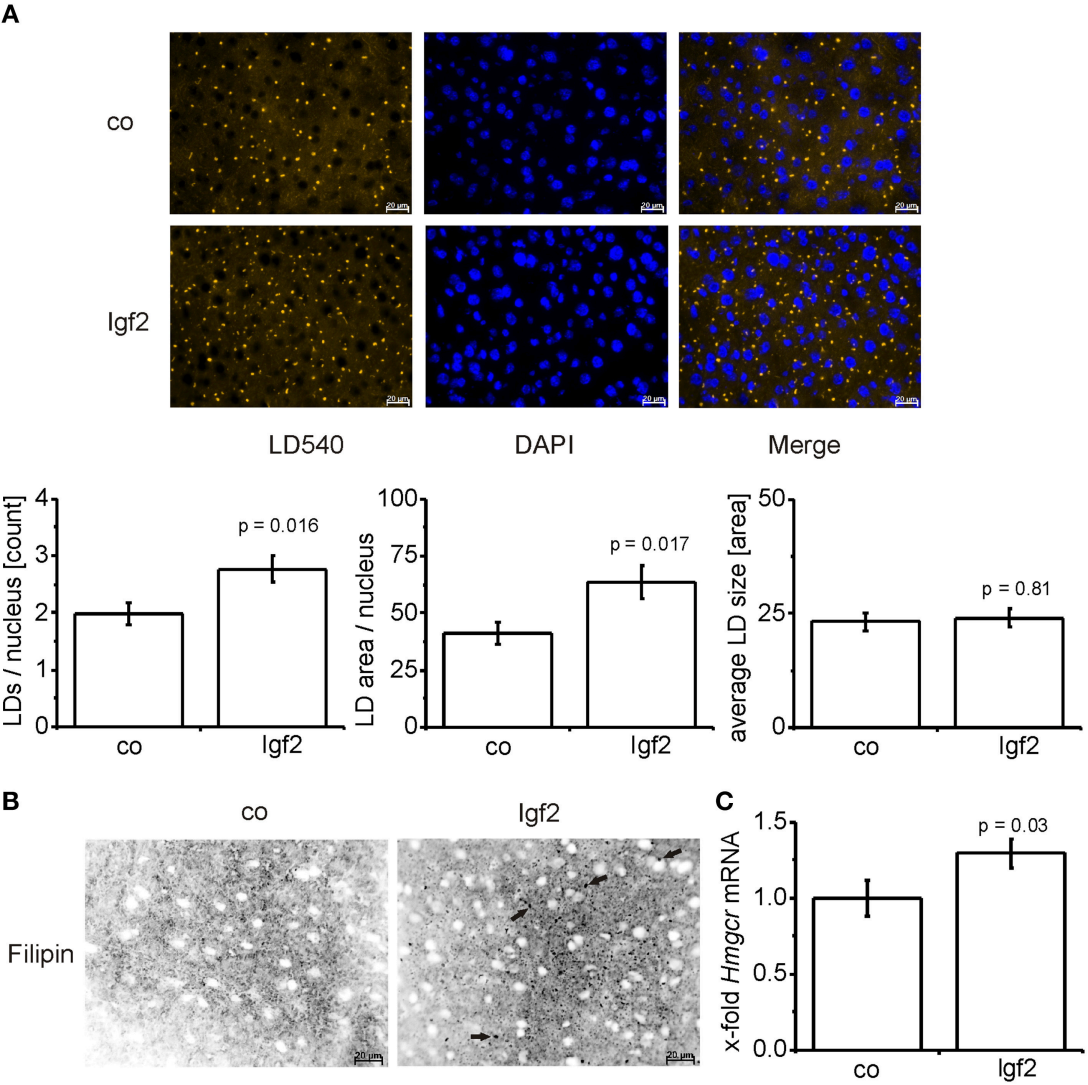
**IGF2 induces lipid droplet formation but has no influence on lipid droplet size. (A)** Representative LD540 fluorescence staining for lipid droplets (LD, yellow droplets) in animals injected with Igf2 plasmid (Igf2) compared to the control plasmid (co), nuclei were stained with DAPI (blue) (scale bar: 20 μm) (upper panel). Five randomly selected pictures per sample were collected and analyzed with ImageJ. Counted LDs were normalized to the nuclei count (lower panel, **left**). Total LD area was normalized to the nuclei count (lower panel, **middle**). Total LD size was estimated as the mean area of all counted LDs (lower panel, **right**) (co, *n* = 10; Igf2, *n* = 11, independent two-sample *t*-test). **(B)** Representative filipin fluorescence staining for free cholesterol (black droplets) in Igf2 and co animals (co, *n* = 10; Igf2, *n* = 11, arrows designate examples for free cholesterol). **(C)** Levels of *Hmgcr* mRNA, real-time RT-PCR (co, *n* = 10; Igf2, *n* = 11, Wilcoxon rank sum test). Data were normalized to *18S* mRNA and co. All results are presented as mean ± SEM.

Since phospholipid levels were elevated and phospholipids are mainly incorporated into the lipid droplet coat and into cell membranes, we analyzed lipid droplet size and number. Lipid droplet staining with LD540 revealed an increased number of lipid droplets in the livers of Igf2 injected mice, but the lipid droplet size showed no distinct alteration (Figure 4A). The livers of Igf2 injected mice also showed a higher amount of free cholesterol visualized with filipin staining (Figure 4B). Concordantly, the mRNA level of the key enzyme involved in cholesterol biosynthesis, hydroxy-methyl-glutaryl-coenzyme A reductase (*Hmgcr*) (Lonardo and Loria, 2012), was upregulated in livers of Igf2 injected animals (Figure 4C).

### *Igf2* effects on transcriptional regulators of lipogenesis

**Figure 5:**
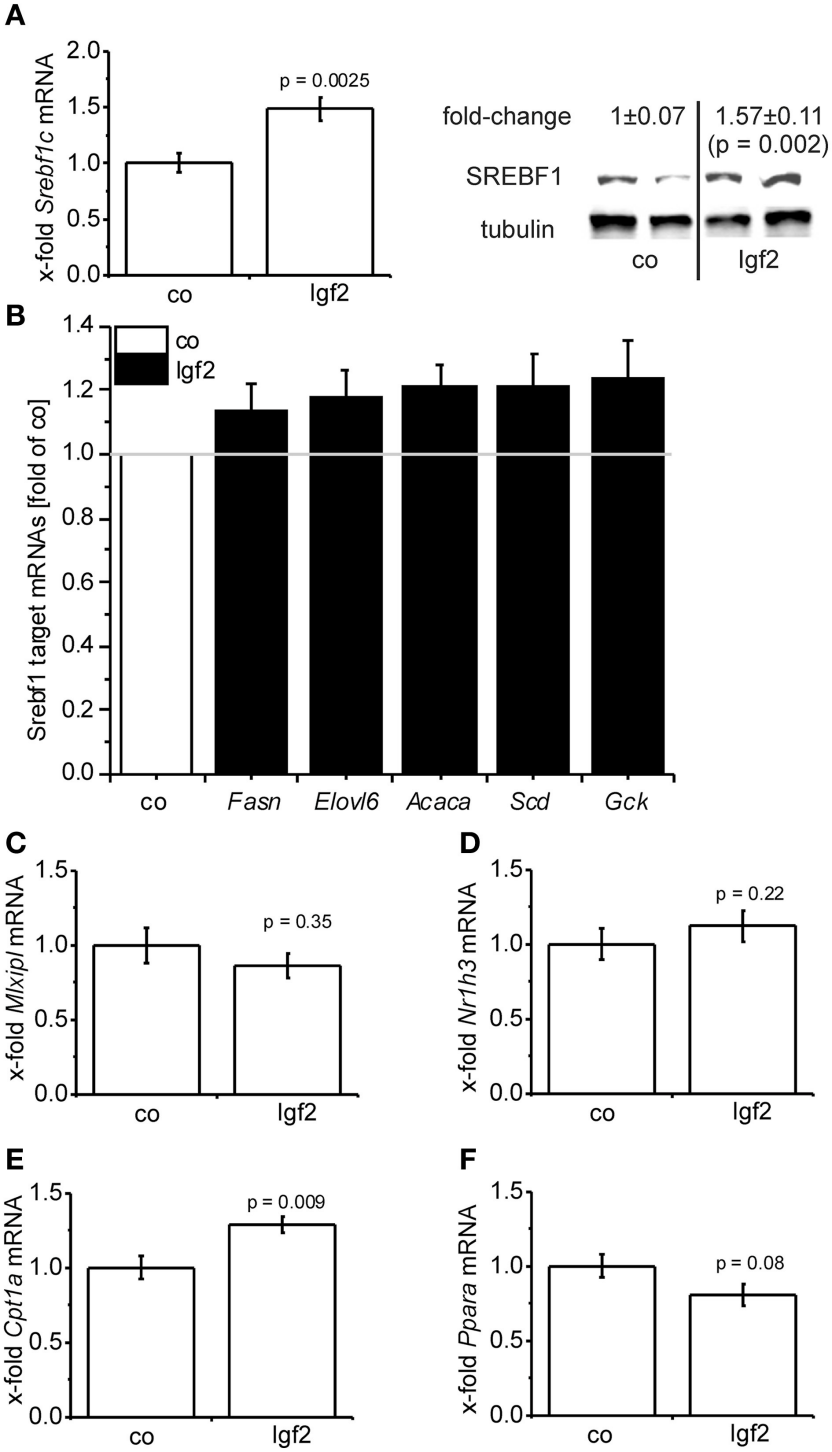
**Igf2 induces lipogenesis. (A)** Lipogenic transcription factor *Srebf1c* mRNA **(left)** and SREBF1 protein **(right)** compared to the control plasmid (co). Left: real-time RT-PCR data (co, *n* = 10; Igf2, *n* = 11, independent two-sample *t*-test) were normalized to *18S* mRNA and co. Right: Western blot analysis of SREBF1 (co, *n* = 8; Igf2, *n* = 9, independent two-sample *t*-test), densitometric data were normalized to α-tubulin and co. **(B)** Expression of SREBF1 target genes *Fasn, Elovl6, Acaca, Scd, Gck* determined by real-time RT-PCR (co, *n* = 10; Igf2, *n* = 11), normalized to *18S* mRNA. Data are shown as fold of co. (c-f) *Mlxipl* **(C)**, *Nr1h3* **(D)**, *Cpt1* **(E)**, *Ppara* **(F)** mRNA expression, determined by real-time RT-PCR data (co, *n* = 10; Igf2, *n* = 11, independent two-sample *t*-test), normalized to *18S* mRNA and co. All results are presented as mean ± SEM.

Igf2 injection induced the expression of the lipogenic transcription factor SREBF1 on mRNA (Figure 5A) and protein (Figure 5A) level. Accordingly, a number of direct transcriptional targets of SREBF1 were slightly induced (Figure 5B). The glucose dependent transcriptional regulator of lipogenic genes carbohydrate response element binding protein (Mlxipl) (Postic and Girard, 2008) was not affected (Figure 5C) by the Igf2-induced activation of the insulin pathway. Interestingly, the gene expression of the main transcriptional regulator of *MLXIPL* and *SREBF1* itself, liver-X-receptor alpha (Nr1h3), was not regulated by Igf2 (Figure 5D). Igf2 injection induced the expression of carnitine palmitoyltransferase 1A (Cpt1a) mRNA (Figure 5E), which is responsible for mitochondrial fatty acid oxidation (Reddy and Rao, 2006), and had rather no effect on mRNA of the peroxisomal fatty acid oxidation inducing peroxisome proliferator activated receptor alpha (Ppara) (Reddy, 2001) (Figure 5F).

## Discussion

IGF2 has been described to be overexpressed in cirrhosis and HCC (Iizuka et al., 2002; Sedlaczek et al., 2003; Couvert et al., 2008; Kessler et al., 2013). Interestingly, despite two studies describing elevated *IGF2* in human steatosis and steatohepatitis (Chiappini et al., 2006) and elevated *Igf2* in murine steatosis (Tybl et al., 2011) and one study showing serum IGF2 inversely correlating with NAFLD-related fibrosis, functional implications of IGF2 in steatosis development are completely unknown. In contrast to IGF2, IGF1 was shown to be downregulated in steatosis (Völzke et al., 2009; Mallea-Gil et al., 2012). Whether the observed downregulation of IGF1 in steatotic patients has a functional role in steatosis development, however, has not been evaluated sufficiently in the literature. To our knowledge, only one study showed that IGF1 can antagonize growth hormone deficiency-induced steatosis (Nishizawa et al., 2012). Up to now it is not completely clear, what distinguishes the effects of IGF2 from the ones of IGF1 in steatosis. Still, the two IGFs show different receptor binding affinities for the IGF and insulin receptors (reviewed in White, 2003, 2006) and therefore signaling can be different. Furthermore, the different expression of both IGFs during development (reviewed in Nakae et al., 2001) also points to specific actions of IGF1 or IGF2, respectively.

Our model of transient hepatic overexpression of IGF2 revealed a causal link of IGF2 overexpression and lipid accumulation. The lack of any zonation in this model is in line with findings in a model of *Igf2* mRNA binding protein 2-2 transgenic mice (Tybl et al., 2011), in which the lipid accumulation is IGF2-dependent (Laggai et al., 2014). When compared with other vector-mediated gene delivery systems, such as viral systems, transient overexpression by hydrodynamic gene delivery has the advantage of being a simple method without the requirement of long and laborious preparation of virus or any special gene transfer device. Furthermore, complications known from adenoviral vectors, such as host immune responses and loss of transfected cells, can be avoided (Zhu et al., 2006).

Interestingly, especially phospholipids were increased in IGF2 overexpressing livers. The LD membrane consists of a phospholipid monolayer, in which phosphatidylcholine is the most prominent phospholipid (Leber et al., 1994). The number of LDs of various sizes has been reported to be increased in murine steatosis (Kochan et al., 2013). Acute steatosis is characterized by rather small LDs, which maturate in chronic steatosis (Pawella et al., 2014).

Also serum levels of phospholipids have been demonstrated to be elevated in NASH patients (Anjani et al., 2015), but were also reported to be decreased during NAFLD progression (Puri et al., 2007), and discussed to have hepato-protective effects (Chamulitrat et al., 2009). Beside the cytoprotective effect of IGF2 the increased phospholipids might be a reason for the lack of an increase in transaminases, since NAFLD is usually accompanied by an increase in transaminases, especially ALT. However, significant liver disease can exist with transaminase levels in the normal range (Papandreou et al., 2007).

LDs were reported to contain high levels of free cholesterol in adipocytes (Prattes et al., 2000). In line with this finding IGF2 overexpressing livers showed increased free cholesterol levels. Steatosis is defined by an accumulation of fat in the liver, mostly in the form of triglycerides (Cohen et al., 2011), but also several other lipid classes, e.g., cholesterol and phospholipids, are stored (Alkhouri et al., 2009). Interestingly, accumulation of triglycerides is not needed for the development of NASH *per se* and might have a rather protective effect (McClain et al., 2007; Yilmaz, 2012). The accumulation of free cholesterol was suggested to play a critical role in NASH development (Tomita et al., 2014) and was shown to positively correlate with the severity of NASH (Caballero et al., 2009; Van Rooyen et al., 2011; Min et al., 2012; Ioannou et al., 2013; Simon et al., 2014b; Tomita et al., 2014). Diet-induced hepatic cholesterol uptake was shown to specifically increase hepatic cholesterol, but not triglycerides (Marí et al., 2006). The latter study underlines that free cholesterol accumulation, but not triglycerides or free fatty acid storage, plays a key role in sensitizing the liver to the development of hepatic inflammation.

Since both the key enzyme in cholesterol biosynthesis, *HMG-CoAR*, and blood cholesterol levels were elevated in IGF2 overexpressing animals, we suggest that cholesterol accumulation in these animals is due to increased *de novo* synthesis of cholesterol. HMG-CoAR is usually induced by SREBF2, but can also be upregulated by SREBF1 (Horton et al., 1998). *De novo* lipid synthesis is regulated in a complex interplay induced by a set of lipogenic transcription factors, such as liver X receptor alpha (LXR-α/NR1H3), sterol regulatory element binding transcription factor 1 (SREBF1/SREBP1), and carbohydrate responsive element binding protein (ChREBP/MLXIPL) (Musso et al., 2009). IGF2 as well as IGF1 and insulin treatment have been shown to induce SREBF1 *via* activation of the insulin and IGF1 receptor (Smith et al., 2008; Laggai et al., 2014). SREBF1 target genes, which are also involved in *de novo* lipid synthesis and steatosis development (Postic and Girard, 2008), were slightly upregulated in IGF2 overexpressing mice supporting lipogenic actions of IGF2. One of the most relevant inducers of lipid degradation in the liver is PPARa (Lefebvre et al., 2006), which was downregulated in IGF2 overexpressing mice. Upregulation of lipolytic Cpt1a, as also observed in IGF2 overexpressing livers, has been described as a compensatory mechanism, which is not able to prevent steatosis (Monetti et al., 2007; Monsénégo et al., 2012).

Taken together, our data show that hepatic IGF2 overexpression can lead to an increased lipid droplet formation and free cholesterol accumulation and might therefore play a causal role in steatosis initiation.

## Author contributions

SL performed most of the experiments. EW, RV, and CL performed the hydrodynamic gene delivery. SL and MO planned and performed the cloning. KG and RM performed GC-MS measurements. JH did the histological analysis. SK, SL, and AK planned experiments, analyzed data, and wrote the manuscript. AK designed and directed the study. All authors critically revised the work, approved the final version of the manuscript to be published, and agreed to be accountable for all aspects of the work.

## Funding

The project was funded, in part, by the Else Kröner-Fresenius-Stiftung (2012_A250 to AK and SK) and the Graduiertenförderung of Saarland University (to SL).

### Conflict of interest statement

The authors declare that the research was conducted in the absence of any commercial or financial relationships that could be construed as a potential conflict of interest.
