# Corrigendum: Transient Hepatic Overexpression of Insulin-Like Growth Factor 2 Induces Free Cholesterol and Lipid Droplet Formation

**DOI:** 10.3389/fphys.2016.00328

**Published:** 2016-07-29

**Authors:** Sonja M. Kessler, Stephan Laggai, Elien Van Wonterghem, Katja Gemperlein, Rolf Müller, Johannes Haybaeck, Roosmarijn E. Vandenbroucke, Manfred Ogris, Claude Libert, Alexandra K. Kiemer

**Affiliations:** ^1^Department of Pharmacy, Pharmaceutical Biology, Saarland UniversitySaarbrücken, Germany; ^2^Inflammation Research Center, VIBGhent, Belgium; ^3^Department of Biomedical Molecular Biology, Ghent UniversityGhent, Belgium; ^4^Department of Microbial Natural Products, Helmholtz Institute for Pharmaceutical Research Saarland, Helmholtz Centre for Infection Research and Pharmaceutical Biotechnology, Saarland UniversitySaarbrücken, Germany; ^5^Institute of Pathology, Medical University of GrazGraz, Austria; ^6^Department of Pharmaceutical Chemistry, University of ViennaVienna, Austria

**Keywords:** insulin-like growth factor 2 (IGF2), NASH, hydrodynamic gene delivery, fatty liver, lipid droplets

Reason for Corrigendum:

In the original article, the name of the third author was mistakenly shortened to Elien van Wonterg. The correct name should be Elien Van Wonterghem instead of Elien Van Wonterg. The authors apologize for this miscommunication.

This error does not change the scientific conclusions of the article in any way.

The original article has been updated.

## Author contributions

Original Work: SL performed most of the experiments. EW, RV, and CL performed the hydrodynamic gene delivery. SL and MO planned and performed the cloning. KG and RM performed GC-MS measurements. JH did the histological analysis. SK, SL, and AK planned experiments, analyzed data, and wrote the manuscript. AK designed and directed the study. All authors critically revised the work, approved the final version of the manuscript to be published, and agreed to be accountable for all aspects of the work. Corrigendum: SL, SK, and AK wrote the Corrigendum.

## Funding

The project was funded, in part, by the Else Kröner-Fresenius-Stiftung (2012_A250 to AK and SK) and the Graduiertenförderung of Saarland University (to SL).

### Conflict of interest statement

The authors declare that the research was conducted in the absence of any commercial or financial relationships that could be construed as a potential conflict of interest.

